# Commentary to: Masoli et al. Clinical Outcomes of CADASIL-Associated NOTCH3 mutations in 451,424 European Ancestry Community Volunteers*.* (Translational Stroke Research Oct 2018)

**DOI:** 10.1007/s12975-018-0681-4

**Published:** 2018-12-18

**Authors:** J. W. Rutten, E. B. van den Akker, S. A. J. Lesnik Oberstein

**Affiliations:** 1 Department of Clinical Genetics, Leiden University Medical Center, 2333 ZA Leiden, The Netherlands; 2 Department of Molecular Epidemiology, Leiden University Medical Center, Leiden, The Netherlands

Dear Editor,

We read with great interest the study by Masoli et al. in which the incidence of cerebrovascular outcomes in individuals with a CADASIL-associated *NOTCH3* mutation in the UK Biobank (UKB) volunteer study is described [1]. Through imputation of array data and subsequent filtering on imputation quality and putative impact on protein function, two *NOTCH3* variants were nominated for further study, namely p.Arg1231Cys and p.Ala1020Pro, with a prevalence of respectively 0.04% and 0.96% in UKB. The p.Arg1231Cys variant was found to be associated with a higher percentage of incident stroke or TIA, whereas p.Ala1020Pro was not. We would like to further discuss several aspects of this important study in the context of recent developments in NOTCH3/CADASIL research.

More than 200 CADASIL-associated *NOTCH3* missense variants have been described in literature, almost exclusively leading to a cysteine alteration in one of the 34 epidermal growth factor-like repeat (EGFr) domains of the NOTCH3 protein [2]. The p.Ala1020Pro variant does not fall into this category and, moreover, has repeatedly been described as a non-pathogenic variant [2–4]. Hence, it is reassuring, though not surprising, that Masoli et al. found that this variant is not associated with increased risk of incident stroke or TIA.

This leaves the p.Arg1231Cys variant as the only detected CADASIL-associated variant in UKB, with a prevalence of 0.4 in 1000. The p.Arg1231Cys variant is located in EGFr domain 31 of the NOTCH3 protein and has been reported as causative in multiple CADASIL pedigrees [5, 6]. We have recently described that CADASIL patients with a cysteine-altering variant in one of EGFr domains 7-34 have a milder phenotype than patients with a variant in one of EGFr domains 1-6 [7]. The fact that EGFr 7-34 variants are milder likely explains why these variants predominate in the general population, whereas the more severe EGFr 1-6 mutations predominate in CADASIL pedigrees. The data of Masoli et al. are in line with this, as they describe that individuals with the p. Arg1231Cys variant had a substantially increased risk of incident stroke or TIA, but nonetheless the number of incident strokes or TIAs during follow-up is much lower than would be expected in CADASIL patients [8].

Furthermore, we would also like to delineate our concerns with respect to the suitability of imputed array data for the detection and subsequent analyses of rare variants. Array data predominantly target variants that are common in the general population. Imputation approaches use these common variants to predict the genotypic status of neighbouring variants not directly measured by the array. Hence, this implies that the genotypic status of rare variants in imputed data is generally the result of a prediction, rather than of a direct measurement. The prediction of the correct genotypic status becomes increasingly difficult with increasing rarity of the variant, as was recently illustrated by Mitt et al. using a study of 500 individuals with HRC imputed array data and directly measured genotypes coming from Whole Exome Sequencing (WES) data. While 17.0% of the predicted genotypes of rare variants (population frequency of ≤ 0.5%.) was false positive, a staggering 58.1% of the directly observed non-reference genotypes in WES were altogether missed by the imputed array data [9]. Comparisons between the results of Masoli et al. and gnomAD, a genome variant database containing sequencing data of 141,456 individuals [10], similarly suggests that UKB systematically underreports the prevalence of CADASIL-associated rare variants in *NOTCH3*. The reported prevalence of 0.04% for the only detected CADASIL-associated variant p. Arg1231Cys in the UKB is identical to the prevalence of this specific variant in the European subpopulation in gnomAD [11]. However, in gnomAD, we also reported an additional 41 distinct more rare CADASIL-associated variants with a total prevalence of CADASIL-associated variants of 3.2/1000. Notably, UKB contains the genetic data of more than three times the individuals of gnomAD. Collectively, this suggests that many of the rare variants in UKB have not been detected, due to the fact that imputation of array data was used, rather than exome- or genome sequencing data.

Cognisance of the full-phenotypic spectrum associated with genomic variants is essential to enable a correct interpretation and prognosis for individuals in whom these variants will be detected as ‘incidental’ findings in the era of whole genome- and exome sequencing. CADASIL, as we know it, reflects only 1% of the total number of individuals with a cysteine altering *NOTCH3* variant in the population [11]. In view of this, and in view of the emerging broad phenotypic spectrum of *NOTCH3* cysteine altering variants, we suggest that the term ‘*NOTCH3* disease spectrum’ may be more appropriate, reserving CADASIL for the severe end of this spectrum.

To conclude, the work by Masoli et al. nicely illustrates how large population-based datasets with longitudinal follow-up can shed light on the full phenotypic spectrum of variants previously only known to be associated with highly penetrant severe disease. With the advent of whole genome sequencing to be performed in 50,000 UKB participants, we look forward to the results of directly measured CADASIL-associated variants and their cerebrovascular phenotypes.
